# Fenebrutinib, a Bruton’s tyrosine kinase inhibitor, blocks distinct human microglial signaling pathways

**DOI:** 10.1186/s12974-024-03267-5

**Published:** 2024-10-27

**Authors:** Julie Langlois, Simona Lange, Martin Ebeling, Will Macnair, Roland Schmucki, Cenxiao Li, Jonathan DeGeer, Tania J. J. Sudharshan, V. Wee Yong, Yun-An Shen, Christopher Harp, Ludovic Collin, James Keaney

**Affiliations:** 1 Roche Pharma Research and Early Development, Neuroscience and Rare Diseases Discovery and Translational Area, Roche Innovation Center Basel, F. Hoffmann-La Roche Ltd, Grenzacherstrasse 124, 4070 Basel, Switzerland; 2 Roche Pharma Research and Early Development, Pharmaceutical Sciences, Roche Innovation Center Basel, F. Hoffmann-La Roche Ltd, Grenzacherstrasse 124, 4070 Basel, Switzerland; 3 Hotchkiss Brain Institute and the Department of Clinical Neurosciences, University of Calgary, 3330 Hospital Dr NW, Calgary, AB Canada; 4 Genentech, Inc., 1 DNA Way, South San Francisco, CA USA

**Keywords:** Multiple sclerosis, Fenebrutinib, BTK, Neuroinflammation, Microglia, Organoids

## Abstract

**Background:**

Bruton’s tyrosine kinase (BTK) is an intracellular signaling enzyme that regulates B-lymphocyte and myeloid cell functions. Due to its involvement in both innate and adaptive immune compartments, BTK inhibitors have emerged as a therapeutic option in autoimmune disorders such as multiple sclerosis (MS). Brain-penetrant, small-molecule BTK inhibitors may also address compartmentalized neuroinflammation, which is proposed to underlie MS disease progression. BTK is expressed by microglia, which are the resident innate immune cells of the brain; however, the precise roles of microglial BTK and impact of BTK inhibitors on microglial functions are still being elucidated. Research on the effects of BTK inhibitors has been limited to rodent disease models. This is the first study reporting effects in human microglia.

**Methods:**

Here we characterize the pharmacological and functional properties of fenebrutinib, a potent, highly selective, noncovalent, reversible, brain-penetrant BTK inhibitor, in human microglia and complex human brain cell systems, including brain organoids.

**Results:**

We find that fenebrutinib blocks the deleterious effects of microglial Fc gamma receptor (FcγR) activation, including cytokine and chemokine release, microglial clustering and neurite damage in diverse human brain cell systems. Gene expression analyses identified pathways linked to inflammation, matrix metalloproteinase production and cholesterol metabolism that were modulated by fenebrutinib treatment. In contrast, fenebrutinib had no significant impact on human microglial pathways linked to Toll-like receptor 4 (TLR4) and NACHT, LRR and PYD domains-containing protein 3 (NLRP3) signaling or myelin phagocytosis.

**Conclusions:**

Our study enhances the understanding of BTK functions in human microglial signaling that are relevant to MS pathogenesis and suggests that fenebrutinib could attenuate detrimental microglial activity associated with FcγR activation in people with MS.

**Supplementary Information:**

The online version contains supplementary material available at 10.1186/s12974-024-03267-5.

## Background

Bruton’s tyrosine kinase (BTK) is a cytoplasmic Tec family tyrosine kinase that is expressed in various immune cell subsets. In B cells, BTK mediates signaling through the B-cell antigen receptor and acts as a crucial regulator of B-cell proliferation, maturation and activation [1]. As such, small-molecule BTK inhibitors have been developed and approved to treat B-cell malignancies linked to dysfunctional B-cell antigen receptor signaling, including mantle cell lymphoma and chronic lymphocytic leukemia [2, 3]. BTK is also expressed in myeloid cells, but our understanding of BTK functions in innate immunity is less advanced. BTK has been associated with signaling through immunoglobulin (Ig)-binding Fc receptors, including Fc gamma receptors (FcγR) in monocytes and macrophages, and Fc epsilon receptors in mast cells [4, 5]. Additionally, BTK is involved in other innate immune pathways such as Toll-like receptor (TLR) signaling and NACHT, LRR and PYD domains-containing protein 3 (NLRP3) inflammasome activation [6–9]. Due to its involvement in both innate and adaptive immune compartments, BTK has emerged as a promising therapeutic target in autoimmune disorders associated with maladaptive B-cell and myeloid cell responses such as rheumatoid arthritis, systemic lupus erythematosus and multiple sclerosis (MS). A number of BTK inhibitors have been tested or are in active clinical development for these indications [10, 11].

MS is a chronic, debilitating central nervous system (CNS) disorder characterized by inflammation, demyelination and neurodegeneration and is the most common cause of nontraumatic neurological disability in young adults [12]. Infiltrating B cells and T cells are crucial components of MS disease pathogenesis and likely contribute to lesion formation and nerve damage via diverse mechanisms, including secretion of inflammatory mediators and production of autoreactive antibodies [13, 14]. Due to their efficacy in reducing relapses and lessening disability progression, anti-CD20 B-cell-depleting therapies are currently used in MS clinical practice and further support the rationale of blocking B-cell functions via BTK inhibition. The potential of BTK inhibitors in MS has also been driven by the emerging role of microglia, the CNS innate immune cells, in compartmentalized neuroinflammation and neurodegeneration [15, 16]. While microglia play a crucial role in maintaining brain homeostasis and responding to tissue damage, detrimental microglial activity may contribute to MS pathogenesis. Iron-positive microglia and macrophages can be found in the demyelinating rims of chronic active lesions that characterize progressive forms of MS [17]. The precise mechanisms by which microglia and macrophages contribute to myelin and neuronal damage are unclear but may involve release of cytokines, chemokines, reactive oxygen species and complement factors. Importantly, microglia and infiltrating monocytes/macrophages express BTK [18–20]. One central feature of MS is the presence of oligoclonal IgG bands in the cerebrospinal fluid (CSF), which are associated with increased cortical lesion load and worsening disability [21, 22]. Furthermore, histopathologic studies have shown that Ig deposition and increased microglial FcγR immunoreactivity characterizes active demyelinating lesions [23, 24], raising the possibility that IgG autoantibodies in the brains of people with MS may induce damaging microglial inflammation via FcγR activation.

Fenebrutinib is a highly potent, selective, brain-penetrant BTK inhibitor [25] currently being tested in three Phase 3 clinical trials (FENhance 1 in people with relapsing multiple sclerosis [pwRMS; NCT04586010] [26], FENhance 2 in pwRMS [NCT04586023] [27] and FENtrepid in people with primary-progressive multiple sclerosis [pwPPMS; NCT04544449] [28]) to establish the effectiveness and safety of fenebrutinib versus standard treatments. In the context of MS it is thought that both peripherally derived B cells and myeloid cells as well as CNS resident myeloid cells including microglia may play a role in compartmentalized inflammation that may be inhibited by fenebrutinib. Unlike BTK inhibitors used to treat B-cell malignancies and most other BTK agents in clinical development for MS, fenebrutinib is noncovalent and has a reversible mode of inhibition [25]. Fenebrutinib reduces disease severity and spinal cord microglial activation in the experimental autoimmune encephalomyelitis mouse model [29]. Other BTK inhibitors recently tested in preclinical rodent models of MS have reported effects on microglial transcriptomic signatures [30]. However, it is challenging to deconstruct the contribution of B-cell versus microglia modulation on the therapeutic benefit in such models, and important differences (e.g. immunometabolic responses) between mouse and human microglia have previously been reported [31–33]. Here we characterize the pharmacological and functional properties of fenebrutinib in human microglia and complex multicellular human systems, including brain tricultures and immunocompetent human brain organoids (IMHBOs), to enhance potential clinical translatability. We have focused our investigations on putative BTK-associated innate immune pathways, including FcγR, TLR and NLRP3 signaling, to understand which components of the human microglial inflammatory response may be modulated by fenebrutinib treatment.

## Methods

### Cell culture

In compliance with Swiss legislation and ethical guidelines, all procedures involving human induced pluripotent stem cells (iPSCs) and their derivatives were meticulously followed. Frozen human iPSC-derived iCell microglia (iMicroglia, R1131) were obtained from Fujifilm Cellular Dynamics Inc. iMicroglia are generated based on protocols described by Abud et al. [34] and are derived from a male, apparently healthy , Caucasian donor whose iPSCs are reprogrammed from a blood sample taken during age range 50–59 y. iMicroglia were thawed based on the supplier protocol in complete iCell Microglia Complete Maintenance Medium (CMM). The CMM media contains 50 mL iCell Glial Base Medium, 0.5 mL iCell Microglia Supplement A (100×), 0.5 mL iCell Microglia Supplement B (100×) and 1 mL iCell Neural Supplement C (50×). After counting, live cells were resuspended in CMM to obtain the desired plating density for treatment and seeded into poly-d-lysine (PDL)-coated plates, as described below. Neurogenin 2-inducible neurons (iNGN2) were generated from the SBAD3 iPSC line (StemBANCC) according to Peitz et al. [35]. Frozen iPSC-derived iCell astrocytes (iAstrocytes, R1092) were obtained from Fujifilm Cellular Dynamics Inc. iAstrocytes are derived from a female, apparently healthy, Caucasian donor whose iPSCs are reprogrammed from fibroblasts at < 18 years old. Cell cultures were maintained at 37 °C with 5% CO_2_ for the indicated time and observed regularly using the Zeiss Primovert inverted microscope.

### Brain tricultures

Brain tricultures were performed with 96-well plates (Thermo Fisher, 165305) that were coated with dendritic polyglycerol amine (100 µg/mL, overnight; Dendrotek, DND400.50-10) and laminin (10 µg/mL, 2 h; Sigma, L2020). On day 1, neurobasal medium (NBM; Gibco, 21103-49) supplemented with B27 (Thermo Fisher, 12587010), GlutaMAX (Thermo Fisher, 35050061), penicillin–streptomycin (Thermo Fisher, 15140122), brain-derived neurotrophic factor (10 ng/mL; Invitrogen, RP-8642), doxycycline (0.4 ng/mL; Sigma, D9891-1G) and ROCK inhibitor (10 µM; Y-27632 Tocris, 1254) was added to each well (50 µL per well). iAstrocytes and iNGN2 (induction day 3) were thawed and seeded into the coated plate (0.5 × 10^4^ iAstrocytes/well and 1.5 × 10^4^ iNGN2/well; 50 µL per well). After 1 h, all media were exchanged with NBM containing all supplements except ROCK inhibitor. On day 3, all media were exchanged with supplemented NBM containing laminin (1 µg/mL; Roche, 11243217001) and ara-C (0.5 µM; Sigma, C6645-25MG). On day 5, iMicroglia were thawed and plated at 1.5 × 10^4^ cells/well in 1:1 supplemented NBM and CMM. On days 7, 10 and 12, 50% media exchanges were performed. All treatments were added on day 15.

### Neural stem cell culture and generation of IMHBOs

The iPSC line BIONI037-A was differentiated into neural stem cells (NSCs), expanded, quality controlled and biobanked as per established protocols [36]. Culture conditions for all cells were 37 °C with 5% CO_2_, and the media used are detailed in the Supplementary file. NSCs were cultured to 70% to 90% confluence in NBM, and accutase was used to detach NSC cultures. To initiate organoid formation, AggreWell 800 plates (StemCell Technologies) were prepared according to the manufacturer’s instructions. Detached NSCs were resuspended in fresh NBM and adjusted to a concentration of 1.5 × 10^6^ cells/mL. This suspension was added to the AggreWell plates containing 1 mL of NBM, centrifuged at 100×*g* for 3 min and incubated for 48 h. Next, 1 mL of media was removed, 1.5 mL of fresh brain organoid medium was added and the organoids were transferred to 6-well plates (Corning, 3471) on a gyratory shaker at 88 rpm. Media changes were performed every other day. For the integration of human iMicroglia into 8-week-old human brain organoids, individual organoids were transferred into 96-well round bottom plates (Corning, 7007) in brain organoid medium supplemented with macrophage colony-stimulating factor. Cell suspensions of iMicroglia were prepared in the same medium with a final concentration of 2 × 10^3^ iMicroglia/organoid and centrifuged to facilitate iMicroglia settling. The plates were incubated for ≥ 1 week, with media changes every other day.

### FcγR activation paradigms

Heat-aggregated IgG (Agg IgG) was prepared by resuspending human IgG (Sigma, I4506) in culture media and heating at 63 °C for 75 min. Agg IgG (1 mg/mL) was added to cells, and supernatants were collected after 24 h. Human IgG (300 µg/mL) was immobilized by coating plates overnight at 37 °C, followed by washing with 1X Dulbecco’s phosphate-buffered saline (PBS). Cells were pretreated with fenebrutinib (0.05 nM to 10 µM) for 30 min prior to seeding in immobilized IgG-containing wells, and supernatants were collected after 24 h.

### iMicroglia-conditioned media-exposed iNGN2

iMicroglia were seeded at 2.8 × 10^5^ cells/well in PDL-coated 24-well plates (Greiner, 662940), pretreated with fenebrutinib (1 µM) or vehicle control for 30 min, then stimulated with Agg IgG for 24 h. Supernatants were collected, centrifuged at 500×*g* for 5 min and stored at − 80 °C before being added to iNGN2. iNGN2 (induction day 3) were thawed and seeded into plates coated with dendritic polyglycerol amine and laminin. iNGN2 were induced for 7 days before being exposed to the iMicroglia-conditioned media (MCM) for 10 days with half media exchange every 2 to 3 days.

### NLRP3 activation

iMicroglia were seeded at 5 × 10^4^ cells/well in PDL-coated 96-well plates (Greiner, 655946). Cells were stimulated with lipopolysaccharide (LPS from E. coli, Serotype O55:B5, Enzo Life Sciences, ALX-581-013; 1 µg/mL) for 3 h. NLRP3 inhibitor tool compound MCC-950 (CP-456773 sodium salt; Selleckchem, S7809; 0.01–1 µM), fenebrutinib (0.01–1 µM) or vehicle (DMSO; Sigma, D2650) were added prior to nigericin (10 µM; Invivogen, tlrl-nig-5) addition. For cytokine measurements, culture fluids were collected after 3 h, centrifuged at 500×*g* for 5 min to remove cell debris and stored at − 80 °C. Interleukin (IL)-1β levels in supernatants from iMicroglia were measured using a human IL-1β homogeneous time-resolved fluorescence kit (Cisbio, 62HIL1B2PEG) following manufacturer’s instructions.

### Myelin phagocytosis assay

Myelin was purified from mouse brain according to Erwig et al. [37] and conjugated to pHrodo indicator dye. iMicroglia were seeded at 2 × 10^4^ cells/well in PDL-coated 96-well plates (Greiner). Cells were incubated in the presence of fenebrutinib (0.01–1 µM), ibrutinib (0.01–1 µM; MedChemExpress, HY-10997) or vehicle control for 24 h. This was followed by addition of pHrodo-conjugated myelin (0.1 µg). Fluorescence was measured by live cell imaging using the IncuCyte S3 system (Sartorius) housed in a cell culture incubator at 37 °C with 5% CO_2_, and the signal was normalized to cell confluency.

### Cytokine and chemokine profiling

iMicroglia were seeded at 5 × 10^4^ cells/well in PDL-coated 96-well plates (Greiner, 655946). Cells were pretreated with fenebrutinib (1 µM) or vehicle control for 30 min and stimulated with IgG for 24 h. Cytokine and chemokine levels were measured in cell culture supernatants using the Human XL Cytokine Array kit (R&D Systems, ARY022B) and LEGENDplex Human Inflammation and Proinflammatory Chemokine Panels (BioLegend, 740809, 740985, 741158). Tumor necrosis factor (TNF)-ɑ levels were measured using the Human Cisbio homogeneous time-resolved fluorescence kit (Cisbio, 62HTNFAPEG) for iMicroglia supernatants. For organoid and triculture supernatants, the Human TNF-ɑ Quantikine High Sensitivity Enzyme-Linked Immunosorbent Assay (ELISA) Kit (R&D systems, HSTA00E) was used.

### Neurofilament light chain analysis

Neurofilament light chain (NfL) levels in supernatants from MCM-exposed iNGN2 and IMHBOs were measured using the NfL ELISA kit from Uman Diagnostics (20-8002).

### NanoString gene expression analysis

iMicroglia were plated at a density of 1.5 × 10^5^ cells/well in 48-well (Costar, 3548) plates. After 24 h of incubation with fenebrutinib (1 µM) and FcγR activators, the cells were washed with 1X PBS and lysed in 350 µL RLT Buffer (Qiagen, 79216). Ribonucleic acid (RNA) was extracted from cell lysates using the Qiagen RNeasy Mini kit (74104). Hybridization reactions were performed using 64 ng of RNA. After completion of the hybridization reactions in the thermal cycler, the nCounter Prep Station was used as described in the nCounter analysis system user manual (MAN-C0035, NanoString). RCC files were downloaded from the digital analyzer. All data analysis was performed using the nSolver 4.0 software and the nCounter Advanced Analysis tool.

### RNA sequencing

Total RNA was isolated from cell lysates of brain tricultures and IMHBOs using the Qiagen RNeasy Mini kit. RNA samples were quantified using Qubit 4.0 Fluorometer (Life Technologies), and RNA integrity was checked with the Agilent 5300 Fragment Analyzer and RNA kit (Agilent Technologies). RNA sequencing was performed by Azenta Life Sciences/Genewiz (Germany). Briefly, libraries were prepared using the NEBNext Ultra II Directional RNA Library Prep Kit for Illumina (NEB). Sequencing libraries were validated using the NGS Kit on the Agilent 5300 Fragment Analyzer (Agilent Technologies) and quantified using Qubit 4.0 Fluorometer (Invitrogen). The sequencing libraries were multiplexed and loaded on the flow cell on the Illumina NovaSeq 6000 instrument. The samples were sequenced using a 2 × 150 pair-end configuration v1.5. Image analysis and base calling were conducted by the NovaSeq Control Software v1.7 on the NovaSeq instrument. Raw sequence data (.bcl files) generated from Illumina NovaSeq were converted into fastq files and demultiplexed using the Illumina bcl2fastq program version 2.20. One mismatch was allowed for index sequence identification.

### Microglia clustering analysis

Tricultures were generated, as described above, using iCell green fluorescent protein (GFP)^+^ microglia (Fujifilm Cellular Dynamics), which constitutively express GFP throughout the cytosol and enable tracking of cell morphology in real time. Tricultures were pretreated with fenebrutinib (1 µM) or vehicle control for 30 min and stimulated with Agg IgG for 10 days with half media exchange every 2 to 3 days. Microglia clustering was quantified by measuring the total integrated intensity of the GFP signal (GCU × µm^2^/well) using the IncuCyte S3 system (Sartorius).

### Immunocytochemistry

Tricultures and monocultures of iNGN2 were fixed in prewarmed paraformaldehyde (PFA)-sucrose (20%) solution for 30 min at 37 °C, followed by 4% PFA for 10 min at room temperature. After washing in PBS, cells were permeabilized for 5 min with 0.25% Triton X-100/PBS. Nonspecific binding was blocked with 10% normal goat serum (Thermo Fisher, PCN5000) for 1 h. Cells were incubated overnight at 4 °C with the following primary antibodies: anti-ionized calcium-binding adaptor molecule 1 (anti-Iba1, 1:500; Fujifilm Wako Pure Chemical Corporation, 019-19741), anti-glial fibrillary acidic protein (anti-GFAP, 1:500; Sigma, AB5541) and anti-pan-neurofilament (anti-pan-NF, 1:500; BioLegend, 837904). Wells were washed three times in 1X PBS for 15 min each and incubated for 1 h at room temperature with the following secondary antibodies: anti-chicken IgG (Alexa488, 1:1000; Thermo Fisher, A11039), anti-mouse (Alexa647; 1:1000; Thermo Fisher, A21235) and anti-rabbit (Alexa568, 1:1000; Thermo Fisher, A143157). After washing in 1X PBS, DAPI (Thermo Fisher, 62248) was added for 5 min. Wells were washed two times with 1X PBS before acquiring images using the Opera Phenix High Content Screening System.

### Whole-mount clearing and immunofluorescent staining of brain organoids

Brain organoids were collected in 1.5-mL protein low-bind Eppendorf tubes and fixed using 4% PFA for 1 h at room temperature. PFA was removed, and organoids were washed 3 times with PBS and stored at 4 °C until further processing. Staining was performed using the HISTO Buffer Kit (Visikol) and was performed according to the protocol for the Neuronal 3D cell culture MAP2 labeling kit with minor modifications. Primary antibodies (anti-Iba1, Abcam; anti-BTK, Cell Signaling Technology) were incubated at 37 °C overnight, and secondary antibodies were incubated at 37 °C for 1.5 h. PBS was removed and ScaleS4 (see Supplementary file) was added, followed by an overnight incubation at 4 °C. Organoids were transferred into 384-well plates (PhenoPlate, Revvity) and imaged using Opera Phenix High Content Screening System.

### Immunohistochemistry of MS brain specimens

Postmortem frozen human brain tissue from individuals with MS was obtained from The Multiple Sclerosis and Parkinson’s Tissue Bank (Imperial College London). These tissue samples were approved by the Wales Research Ethics Committee as a Research Tissue Bank (Ref. No. 18/WA/0238). The use of these human tissues for research was approved by the Conjoint Health Research Ethics Board at the University of Calgary (Ethics ID REB15-0444). Case MS352, which included a male patient who was aged 43 years at death, was used as an example. Tissue slides were thawed at room temperature for 30 min and rehydrated with PBS before fixing with 4% PFA for 10 min at room temperature. The slides stained for myelin basic protein (MBP) were additionally delipidated with a series of ascending 50%, 70%, 90%, 95% and 100% ethanol washes for 1 min each, followed by descending concentrations of ethanol washes for 1 min each. The tissue was then fixed with 4% PFA for 10 min. After fixation, all slides were washed with PBS before permeabilizing with 0.2% Triton X-100 in PBS for 10 min. The tissue was then blocked with a horse serum solution (0.01 M PBS; 10% horse serum; 1% bovine serum albumin; 0.1% cold fish skin gelatin; 0.1% Triton X-100; 0.05% Tween-20) for 1 h at room temperature. Slides were then incubated with primary antibodies diluted in antibody dilution buffer (0.01 M PBS; 1% bovine serum albumin; 0.1% cold fish skin gelatin; 0.5% Triton X-100) at 4 °C overnight. The primary antibodies used included mouse anti-human BTK (1:100; BD Biosciences, 611117), rat anti-human CD45 (1:500; Invitrogen, MA5-17687), mouse anti-human leukocyte antigen-DR isotype (HLA-DR; 1:500; Invitrogen, 14-9956-82) and chicken anti-human MBP (1:500; Invitrogen, PA1-100008). Slides were washed three times with 0.1% Tween-20 in PBS, then incubated with fluorophore-conjugated secondary antibodies (1:400) and 1 μg/mL of DAPI in antibody dilution buffer for 1 h at room temperature. The slides were washed three times with PBS-Tween and then mounted using Fluoromount G (SouthernBiotech, 0100-01). Stained slides were stored at 4 °C. Images were captured with the Leica TCS SP8 Confocal Laser Scanning microscope using the 10 × 0.4 and 25 × 0.95 numerical aperture water objective lenses. The pinhole was set to 1 airy unit, and higher magnification images were acquired in a z-stack scanning of 15 sections. Fluorophores were excited using 405-nm, 488-nm, 552-nm and 640-nm lasers and detected by two Hamamatsu photomultiplier tube detectors and two hybrid detectors. The same laser power, gain and offset settings were kept consistent for stained sections.

### Western blot

iMicroglia were washed with ice-cold PBS and lysed in RIPA buffer (Sigma-Aldrich, R0278) containing protease and phosphatase inhibitors (Roche, 11697498001, 4906845001). Protein concentration in cell lysates were measured using the Pierce BCA Protein Assay kit (Thermo Fisher, 23225). Equal amounts of protein were separated by gel electrophoresis using 4–20% precast polyacrylamide gels (BioRad, 4561095) and transferred to PVDF membranes (BioRad, 1704156). Nonspecific binding was blocked by incubating membranes for 20 min in blocking buffer (BioRad, 12010020), followed by overnight incubation at 4 °C with the following primary antibodies: rabbit anti-phospho-BTK (1:100; Abcam, ab68217), rabbit anti-BTK (1:1000; Cell Signaling Technology, D3H5) and mouse anti-β-actin conjugated with horseradish peroxidase (1:5000; Abcam, ab20272). Blots were washed three times in 1X TBS with 0.1% Tween-20 (TBS-T), followed by incubation with secondary antibodies conjugated with horseradish peroxidase for 1 h at room temperature. After three additional washes in TBS-T, blots were imaged using the ChemiDoc imaging system and quantified using ImageLab software (BioRad). Full uncropped Western blot images are shown in Supplementary Fig. 1.

### Bioinformatics analysis

Base calling was performed with bcl to fastq file converter bcl2fastq2 version 2.20.0 (BCL to FASTQ file converter, available online at Illumina Inc.). Fastq files were quality checked with FastQC version 0.11.9 from the Babraham Institute. Paired-end reads were mapped onto the human genome (build “hg38”) with read aligner STAR version 2.7.10b using default mapping parameters [38]. Alignment metrics were determined with Picard version 2.25.1 (Picard Toolkit, 2018. Broad Institute, GitHub Repository: http://broadinstitute.github.io/picard/). Read sequences and alignments were quality checked with MultiQC version 1.12 [39]. Numbers of mapped reads for all RefSeq and/or Ensembl transcript variants of a gene were combined into a single value (i.e. read count) assuming a reverse-stranded library by featureCounts version 2.0.1 [40] and normalized as transcripts per million. We applied the edgeR algorithm [41] for differential gene expression analysis. We performed the adapted CAMERA algorithm [42] for gene set enrichment analysis, which was implemented in the ribiosNGS package (next-generation sequencing data analysis with ribios; https://github.com/bedapub/ribiosNGS).

### Statistical analysis

Data and statistical analyses were performed using GraphPad Prism 9.0 software. Results are presented as means ± standard deviation unless otherwise stated. Data were analyzed using two-tailed Student’s t-test to compare between two groups. One-way analysis of variance was performed, followed by Tukey’s post hoc test for multiple comparison testing, with a *P* value of < 0.05 considered statistically significant. Data from the MS lesion single-nuclei RNA sequencing (snRNA-seq) study were analyzed using the R package *glmmTMB* and the Benjamini–Hochberg procedure used to control the false discovery rate [43].

## Results

### BTK and innate immune pathway gene expression in human microglia and across MS lesions

**Fig. 1:**
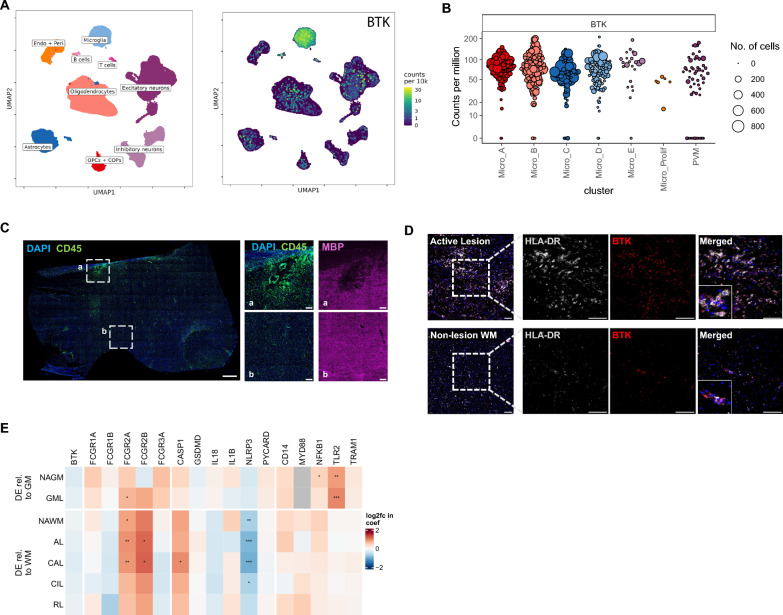
BTK and innate immune pathway gene expression in human microglia and across MS lesion types. **A** Uniform Manifold Approximation and Projection plot showing expression of BTK (right) in an snRNA-seq dataset of human brain samples. BTK is highly expressed in microglia in the human CNS. **B** snRNA-seq dataset showing broad expression of BTK across microglial subclusters from the human brain. Dots correspond to “pseudobulk” values for clusters, i.e. the sum of counts across all cells in one cluster, in one sample. Counts are normalized by the TMM-normalized library size. Values are only shown where there are at least 10 cells in the combination of cluster and sample. **C** Immunofluorescent images of postmortem MS brain tissue from subcortical WM containing an active lesion. Low-magnification overview, with DAPI identifying cell nuclei (blue), CD45 identifying immune cells (green) and MBP identifying myelin (magenta), with dotted white boxes containing higher magnification images showing the active lesion (**a**) and nonlesion WM (**b**). Scale bar is 2 mm. **D** The active lesion area shows prominent infiltration of microglia and macrophages labeled with HLA-DR (white) that are closely associated with BTK staining (red), as shown in the merged panels. Scale bars are 100 μm. **E** Heat map showing expression of BTK and associated innate immune pathway genes in microglia across MS lesion types. Differential expression relative to control WM and GM is shown. Expression of certain FcγR-encoding genes, including *FCGR2A* and *FCGR2B*, is significantly increased in WM active lesions and chronic active lesions. Significance is indicated by ****P < 0.0001, ***P < 0.001, **P < 0.01 and *P < 0.05. R package *glmmTMB* was used, and the Benjamini–Hochberg procedure was used to control the false discovery rate. *AL* active lesion, *BTK* Bruton’s tyrosine kinase, *CAL* chronic active lesion, *CIL* chronic inactive lesion, *CNS* central nervous system, *COP* committed oligodendrocyte precursor, *DAPI* 4′,6-diamidino-2-phenylindole, *DE* differential expression, *endo* endothelial, *FcγR* Fc gamma receptor, *GM* gray matter, *GML* gray matter lesion, *HLA-DR* human leukocyte antigen-DR variant, *MBP* myelin basic protein, *MS* multiple sclerosis, *NAGM* normal-appearing gray matter, *NAWM* normal-appearing white matter, *OPC* oligodendrocyte precursor cell, *peri* pericyte, *prolif* proliferating, *PVM* perivascular macrophage, *RL* remyelinating lesion, *snRNA-seq* single-nuclei ribonucleic acid sequencing, *WM* white matter

In the periphery, BTK is expressed in adaptive immune cells, including B cells and innate immune cells such as monocytes and macrophages. To assess cell type expression of BTK in the human CNS, we leveraged an snRNA-seq dataset of white matter and gray matter samples from controls and individuals with MS [43]. In this dataset, *BTK* was primarily expressed in microglia in the CNS, with some expression in smaller cell populations, including B cells (Fig. 1A). Microglial subclusters have previously been defined in this dataset and include Micro_A and Micro_B, which show relatively high expression of homeostatic markers such as *P2RY12* and *CXCR1* and Micro_C-E, which express higher levels of MHC class II molecule transcripts (e.g. *HLA-DPB1*), *TREM2* and *APOE* [43]. Micro_D and Micro_E most closely map to disease-associated microglia and microglia inflamed in MS, which are microglial subtypes that have been reported in neurodegenerative diseases [44]. We observed that *BTK* was broadly expressed across microglial subclusters (Fig. 1B). To confirm the presence of microglial BTK at the protein level in the brains of individuals with MS, postmortem brain tissue sections were probed for CD45 and MBP to identify areas with lesions. An active lesion was characterized by demyelination (loss of MBP) and an extensive accumulation of CD45^+^ immune cells (Fig. 1C). HLA-DR^+^ microglia and macrophages were prominent in the active lesion, and the majority were immunoreactive for BTK (Fig. 1D). An area of the white matter that did not have an obvious lesion (intact MBP) is shown in Fig. 1C. In contrast to the active lesion, the nonlesion white matter had much fewer BTK^+^ cells, and many HLA-DR^+^ microglia/macrophages were not immunoreactive for BTK (Fig. 1D). Next, we used the snRNA-seq dataset to examine *BTK* expression and the associated innate immune pathway genes in microglia across MS lesion types to understand which, if any, BTK-related pathways may be transcriptionally dysregulated in MS brain tissue. While microglial *BTK* transcript levels were not upregulated in white or gray matter lesion types compared with control tissue, FcγR-encoding genes, including *FCGR2A* and *FCGR2B*, were significantly upregulated in active and chronic active lesions (Fig. 1E). *FCGR2A* was also elevated in normal-appearing white matter and gray matter lesions. Among representative genes of the NLRP3 pathway, *CASP1* only demonstrated significant upregulation in chronic active lesions, whereas expression of other genes was unchanged, and *NLRP3* was significantly downregulated in normal-appearing white matter and white matter lesions (Fig. 1E). Among representative genes of the TLR2/4 pathways, *TLR2* was significantly upregulated in normal-appearing gray matter and gray matter lesions while expression of other genes was unchanged in both white matter and gray matter (Fig. 1E). *TLR4* transcripts were not detected in this snRNA-seq dataset. In summary, this analysis indicates that microglia are a potential cellular target of BTK inhibitors in the human CNS and that expression of specific FcγR-encoding genes is elevated in microglia of active MS lesions.

### Fenebrutinib blocks inflammatory pathways linked to FcγR activation in human iMicroglia

**Fig. 2:**
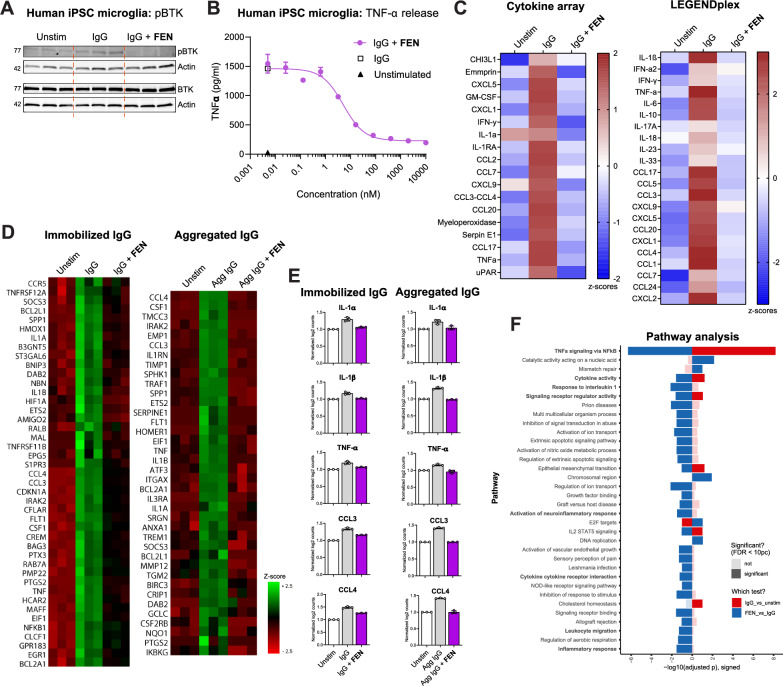
Fenebrutinib blocks inflammatory pathways linked to FcγR activation in human iMicroglia. **A** Western blot of protein lysates from human iMicroglia incubated with fenebrutinib (1 μM) and stimulated with immobilized IgG (300 μg/mL) for 30 min. Fenebrutinib treatment reduced immobilized IgG-induced BTK activation, as shown by pBTK levels. Cropped images show relevant bands for pBTK, BTK and actin. Full uncropped images are shown in Supplementary Fig. 1. **B** TNF-α release from human iMicroglia incubated with fenebrutinib and stimulated with immobilized IgG (300 μg/mL) for 24 h. Fenebrutinib (0.05 nM to 10 µM) demonstrated potent concentration-dependent reduction in immobilized IgG-induced TNF-α release. **C** Extended cytokine and chemokine release profiles from human iMicroglia incubated with fenebrutinib (1 μM) and stimulated with immobilized IgG (300 μg/mL; 24 h) using two different immunoassay methods. Fenebrutinib treatment attenuated the release of multiple proinflammatory cytokines and chemokines. **D** NanoString® gene expression profiling of human iMicroglia incubated with fenebrutinib (1 μM) and stimulated with immobilized IgG (300 μg/mL) or aggregated IgG (1 mg/mL) for 24 h. Fenebrutinib reverses transcriptional signatures associated with FcγR activation. **E** Expression levels of key proinflammatory cytokines (IL-1α, IL-1β and TNF-α) and chemokines (CCL3 and CCL4) are reduced with fenebrutinib treatment. **F** Gene set enrichment analysis identified FcγR activation-induced pathways linked to neuroinflammation, cytokine signaling and inflammatory response (displayed in bold) that are modulated by fenebrutinib treatment. Data are shown as mean ± SD, with three to four replicates per condition. Individual dots in **E** represent replicate wells. Results are representative of two to three independent experiments. *Agg IgG* heat-aggregated immunoglobulin G, *BTK* Bruton’s tyrosine kinase, *CCL* C-C motif chemokine ligand, *CHI3L1* chitinase-3-like protein 1, *CXCL* C-X-C motif chemokine ligand, *DNA* deoxyribonucleic acid, *FcγR* Fc gamma receptor, *FDR* false discovery rate, *FEN* fenebrutinib, *GM-CSF* granulocyte–macrophage colony-stimulating factor, *IFN* interferon, *Ig* immunoglobulin, *IL* interleukin, *iMicroglia* induced pluripotent stem cell-derived microglia, *iPSC* induced pluripotent stem cell, *NOD* nucleotide-binding oligomerization domain, *pBTK* phosphorylated Bruton’s tyrosine kinase, *SD* standard deviation, *STAT5* signal transducer and activator of transcription 5, *TNF* tumor necrosis factor, *uPAR* urokinase-type plasminogen activator receptor

Once microglial expression of BTK-related genes in the human CNS was established, we used human iMicroglia as a tool to assess the impact of fenebrutinib in modulating microglial innate immune pathways. BTK has previously been linked to FcγR signaling in peripheral monocytes and macrophages, and certain FcγR-encoding genes were upregulated in MS lesions in the snRNA-seq dataset. Therefore, we first investigated the effects of fenebrutinib in the context of FcγR activation, using BTK autophosphorylation as a marker of BTK activation. Treatment of human iMicroglia with fenebrutinib reduced phosphorylated BTK levels following immobilized IgG stimulation (Fig. 2A, Supplementary Fig. 2). To assess the efficacy and potency of fenebrutinib in blocking downstream effects of FcγR activation, TNF-α release was measured in human iMicroglia stimulated with immobilized IgG. Fenebrutinib treatment demonstrated a strong reduction in immobilized IgG-induced TNF-α release (Supplementary Fig. 3), with a half-maximal inhibitory concentration (IC_50_) of 5.1 nM (Fig. 2B). We also confirmed that immobilized IgG-induced cytokine release was due to FcγR activation and not related to unspecific binding of IgG to cell surface receptors since Fab fragment did not induce TNF-α release (Supplementary Fig. 4). Extended cytokine and chemokine profiling of human iMicroglia using two different immunoassay methods identified a significant increase in the release of multiple cytokines (e.g. IL-6, interferon gamma, IL-1α and IL-1β) and chemokines (e.g. CCL2, CCL3 and CCL4) following stimulation with immobilized IgG, which was reversed with fenebrutinib treatment (Fig. 2C). NanoString gene expression profiling of human iMicroglia was performed to (1) understand whether elevated cytokine and chemokine release following FcγR activation is due to changes at a transcriptional level and (2) to capture transcriptional signatures associated with fenebrutinib treatment. Activation of FcγR signaling was achieved via immobilized IgG or Agg IgG, having confirmed that Agg IgG also induces FcγR activation (Supplementary Fig. 4). With both approaches, fenebrutinib reversed transcriptional signatures associated with FcγR activation (Fig. 2D). Both immobilized IgG and Agg IgG increased expression of key cytokines (*IL1A*, *IL1B* and *TNF*) and chemokines (*CCL3* and *CCL4*), which were reduced with fenebrutinib treatment (Fig. 2E). Consistent with these findings, pathway analysis identified that FcγR activation induced pathways linked to neuroinflammation and cytokine signaling such as TNF-α signaling via NFκB, response to IL-1 and positive regulation of neuroinflammatory response (Fig. 2F). Thus, fenebrutinib strongly blocks inflammatory responses linked to FcγR activation in human iMicroglia.

### Fenebrutinib blocks effects of FcγR activation in human brain tricultures and MCM-exposed neurons

**Fig. 3:**
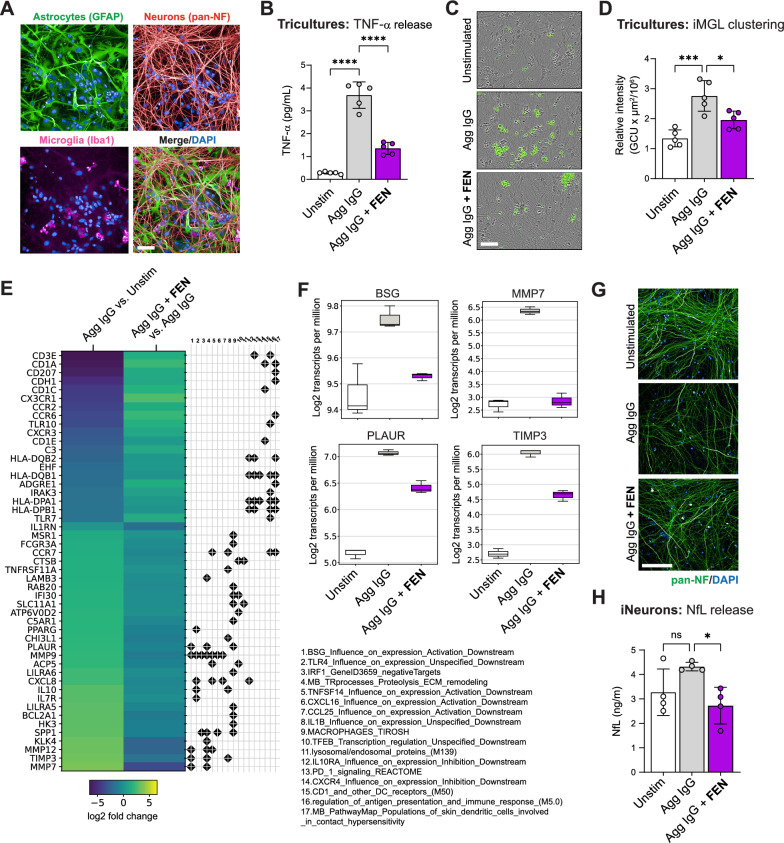
Fenebrutinib blocks effects of FcγR activation in human brain tricultures and MCM-exposed neurons. **A** Immunocytochemistry of the human iPSC-derived brain triculture system showing GFAP^+^ astrocytes, pan-NF^+^ neurons and Iba1^+^ microglia. Scale bar is 50 µm. **B** TNF-α release from human iPSC-derived brain tricultures treated with fenebrutinib (1 μM) and stimulated with Agg IgG (1 mg/mL) for 24 h. Fenebrutinib treatment reduced Agg IgG-induced TNF-α release. **C** Representative live cell microscopy images of human iPSC-derived brain tricultures showing Agg IgG-induced clustering of GFP^+^ iMicroglia. Scale bar is 100 µm. **D** Quantification of GFP^+^ iMicroglia demonstrated a significant reduction in microglial clustering with fenebrutinib treatment. **E** Heatmap showing top differentially expressed genes from annotated gene sets in human iPSC-derived brain tricultures treated with fenebrutinib (1 μM) and stimulated with Agg IgG (1 mg/mL) for 72 h. **F** Box plots showing representative genes from the BSG pathway, which is the most significant gene set modulated by fenebrutinib treatment. **G** Representative confocal images of MCM-exposed iNGN2 showing reduced neurite staining with Agg IgG MCM, which is reversed by fenebrutinib treatment. Scale bar is 200 µm. **H** NfL levels in supernatants of MCM-exposed iNGN2. Fenebrutinib treatment significantly reduced NfL release compared with Agg IgG alone. Data are shown as mean ± SD; individual dots represent replicate wells. Results are representative of two to three independent experiments. Significance is indicated by ****P < 0.0001, ***P < 0.001 and *P < 0.05, determined by one‐way ANOVA and Tukey’s post hoc test. *Agg IgG* heat-aggregated immunoglobulin G, *ANOVA* analysis of variance, *BSG* basigin, *DAPI* 4′,6-diamidino-2-phenylindole, *FcγR* Fc gamma receptor, *FEN* fenebrutinib, *GFAP* glial fibrillary acidic protein, *GFP* green fluorescent protein, *iMicroglia/iMGL* induced pluripotent stem cell-derived microglia, *iNGN2* neurogenin 2-inducible neuron, *Iba1* ionized calcium-binding adaptor molecule 1, *iPSC* induced pluripotent stem cell, *MCM* microglia-conditioned media, *NF* neurofilament, *NfL* neurofilament light chain, *ns* not significant, *SD* standard deviation, *TNF* tumor necrosis factor, *tpm* transcripts per million

To validate and extend the findings in human iMicroglia monocultures to more complex brain cell systems, we investigated the effects of FcγR activation and fenebrutinib treatment in human iPSC-derived brain tricultures consisting of iNGN2, iAstrocytes and iMicroglia (Fig. 3A). Due to technical concerns regarding long-term culturing of iNGN2 and iAstrocytes on immobilized IgG, Agg IgG was selected as the preferred method for FcγR engagement in this system. First, the ability of Agg IgG to activate FcγR signaling in human brain tricultures was assessed by measurement of TNF-α release. Short-term stimulation (24 h) of human brain tricultures with Agg IgG enhanced TNF-α release, which could be significantly reversed with fenebrutinib treatment (Fig. 3B). Interestingly, using GFP^+^ iMicroglia and live cell imaging, we observed that long-term exposure (10 days) to Agg IgG induced significant microglial clustering, which was reduced with fenebrutinib treatment (Fig. 3C, D). Similar microglial clustering has previously been reported following LPS stimulation of human iMicroglia in co-culture with neurons [45] and in the vicinity of active demyelinating lesions in individuals with MS [46]. To understand the impact of FcγR activation in human brain tricultures more broadly, RNA sequencing was performed on Agg IgG-exposed tricultures, and the top differentially expressed genes and annotated gene sets were assessed (Fig. 3E). Both downregulated and upregulated genes mapped to predominantly immune-related gene sets and included those related to pathways involving basigin (BSG), macrophages, CCL25 and IL-1β (all upregulated with Agg IgG stimulation) and lysosomal/endosomal proteins, IL-10 receptor and antigen presentation (all downregulated with Agg IgG). The most significant gene set modulated by fenebrutinib included genes belonging to the BSG pathway (Fig. 3F). BSG (also known as EMMPRIN) is an inducer of matrix metalloproteinases (MMPs), which can contribute to blood–brain barrier (BBB) breakdown [47]. BSG levels are also elevated in the plaques of individuals with MS, and BSG blockade has been shown to reduce clinical severity in experimental autoimmune encephalomyelitis models [48]. Next, to assess the impact of microglial FcγR activation on neuronal integrity in human brain tricultures, we measured neurite density using immunocytochemistry and NfL release using ELISA; however, we did not detect clear changes (Supplementary Fig. 5). Since TNF-α levels were almost 300-fold lower in human brain tricultures than in iMicroglia monocultures (compare Figs. 2B and 3B), we hypothesized that higher cytokine and chemokine concentrations may be required to observe effects on neuronal integrity. To this end, iNGN2 were exposed to MCM from iMicroglia monocultures stimulated with Agg IgG in the presence or absence of fenebrutinib. Reduced neurite staining was observed in iNGN2 exposed to Agg IgG MCM for 10 days, which was reversed with Agg IgG + fenebrutinib MCM exposure (Fig. 3G). A slight increase in NfL release was detected in Agg IgG MCM-exposed iNGN2 and was significantly reduced with exposure to Agg IgG + fenebrutinib MCM (Fig. 3H). In summary, fenebrutinib treatment blocks the effects of FcγR activation in human brain tricultures and improves neuronal integrity following exposure to conditioned media from FcγR-activated iMicroglia.

### Fenebrutinib inhibits downstream pathways linked to FcγR activation and lysolecithin exposure in IMHBOs

**Fig. 4:**
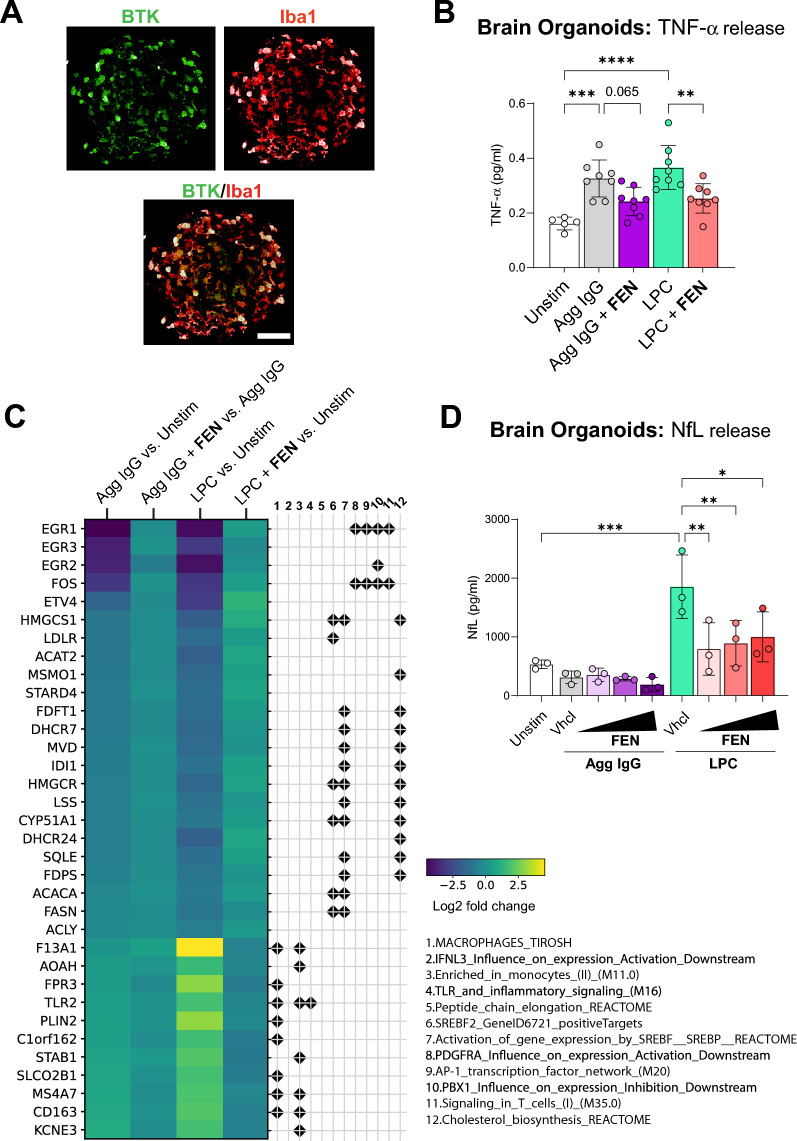
Fenebrutinib inhibits downstream pathways linked to FcγR activation and LPC exposure in IMHBOs. **A** Immunoreactivity of BTK and Iba1 in IMHBOs showing overlap between BTK expression in iMicroglia in IMHBOs. Scale bar is 100 µm. **B** TNF-α release from IMHBOs incubated with fenebrutinib (1 μM) and stimulated with Agg IgG (1 mg/mL; 24 h) or LPC (0.1 mM; 72 h). Fenebrutinib treatment reduced Agg IgG- and LPC-induced TNF-α release. **C** Heatmap showing top differentially expressed genes from annotated gene sets in IMHBOs treated with fenebrutinib (1 μM) and stimulated with Agg IgG (1 mg/mL; 24 h) or LPC (0.1 mM; 72 h). **D** NfL levels in supernatants of IMHBOs incubated with fenebrutinib (0.01–1 μM) and stimulated with Agg IgG (1 mg/mL; 24 h) or LPC (0.1 mM; 72 h). Fenebrutinib treatment significantly reduced LPC-induced NfL release. Data are shown as mean ± SD; individual dots represent replicate wells from single organoids. Significance is indicated by ****P < 0.0001, ***P < 0.001, **P < 0.01 and *P < 0.05, determined by one‐way ANOVA and Tukey’s post hoc test. *Agg IgG* heat-aggregated immunoglobulin G, *ANOVA* analysis of variance, *BTK* Bruton’s tyrosine kinase, *FcγR* Fc gamma receptor, *FEN* fenebrutinib, *Iba1* ionized calcium-binding adaptor molecule 1, *IMHBO* immunocompetent human brain organoid, *iMicroglia* induced pluripotent stem cell-derived microglia, *LPC* lysolecithin, *NfL* neurofilament light chain, *SD* standard deviation, *TNF* tumor necrosis factor

The advent of new 3D systems such as brain organoids allows for investigation of more complex cell–cell interactions that may better mimic the human brain environment [49]. Microglia integrated into organoids have been reported to show a transcriptional shift toward in vivo human microglia relative to 2D microglia in monoculture and to improve neuronal maturation and function [50–52]. To this end, we examined the effects of FcγR activation in IMHBOs comprising iPSC-derived neurons, astrocytes, oligodendrocytes and integrated microglia. The membrane-disrupting agent lysolecithin (LPC) was also applied to IMHBOs, as it has been shown to induce microglial/macrophage infiltration and activation, astrocyte reactivity and axonal injury [53, 54]. First, we used immunocytochemistry to confirm that BTK is expressed in microglia that are integrated into human brain organoids (Fig. 4A). Similar to findings in human iMicroglia monocultures and brain tricultures, stimulation of IMHBOs with Agg IgG increased TNF-α release, which was reduced with fenebrutinib treatment (Fig. 4B). Fenebrutinib also significantly reduced LPC-induced TNF-α release (Fig. 4B). Next, RNA sequencing was performed to assess genes and pathways impacted by exposure of IMHBOs to Agg IgG and LPC (Fig. 4C). Interestingly, both Agg IgG and LPC induced similar gene expression changes, with upregulation of gene sets related to inflammatory signaling (macrophages, interferon λ-3 and TLR) and downregulation of gene sets related to lipid metabolism and cholesterol regulation (sterol regulatory element binding protein [SREBP] and cholesterol biosynthesis). Fenebrutinib treatment largely reversed Agg IgG- and LPC-mediated gene expression changes in IMHBOs (Fig. 4C). NfL release from IMHBOs was measured to investigate the effects of Agg IgG and LPC exposure on neuronal integrity. Agg IgG exposure did not increase NfL release; however, LPC induced a significant elevation in NfL levels in the supernatant, which were reduced with fenebrutinib treatment (Fig. 4D). Thus, downstream effects of FcγR activation and LPC exposure in IMHBOs can be ameliorated with fenebrutinib treatment.

### Human microglial pathways not impacted by fenebrutinib treatment

**Fig. 5:**
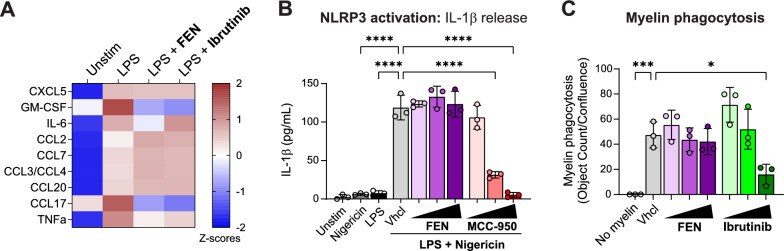
Human microglial pathways not impacted by fenebrutinib treatment. **A** Cytokine and chemokine release from LPS-stimulated (0.1 μg/mL) human iMicroglia incubated with fenebrutinib or ibrutinib (both 1 μM) for 24 h. While BTK inhibition reduced levels of GM-CSF and CCL17, limited effects were observed for other cytokines and chemokines that are increased with LPS stimulation. **B** IL-1β release from human iMicroglia incubated with fenebrutinib or MCC-950 (0.01–1 μM) and stimulated with LPS (0.1 μg/mL; 3 h) followed by nigericin (10 μM; 1 h). Fenebrutinib had no effect on IL-1β release in contrast to MCC-950, which dose dependently reduced IL-1β levels. **C** Phagocytosis of pHrodo-conjugated myelin by human iMicroglia incubated with fenebrutinib or ibrutinib (0.01–1 μM) for 24 h. Fenebrutinib had no effect on myelin phagocytosis, while ibrutinib reduced myelin phagocytosis at 1 μM only. Data are shown as mean ± SD; individual dots represent replicate wells. Results are representative of two to three independent experiments. Significance is indicated by ****P < 0.0001, ***P < 0.001 and *P < 0.05, determined by one‐way ANOVA and Tukey’s post hoc test. *ANOVA* analysis of variance, *BTK* Bruton’s tyrosine kinase, *CCL* C-C motif chemokine ligand, *CXCL* C-X-C motif chemokine ligand, *GM-CSF* granulocyte–macrophage colony-stimulating factor, *FEN* fenebrutinib, *IL* interleukin, *iMicroglia* induced pluripotent stem cell-derived microglia, *LPS* lipopolysaccharide, *NLRP3* NACHT, LRR and PYD domains-containing protein 3, *SD* standard deviation, *TNF* tumor necrosis factor

Inhibition of BTK has previously been implicated in the modulation of innate immune pathways beyond FcγRs, including TLR4 and NLRP3 signaling [8, 55, 56] and functional activities such as phagocytosis [18, 57]. However, to our knowledge, a link between BTK inhibition and these cellular phenotypes in human microglia has not been reported. We first investigated the TLR4 pathway in human iMicroglia via LPS stimulation. In contrast to its effects on FcγR activation, fenebrutinib had limited impact on LPS-induced cytokine and chemokine release in iMicroglia, with only granulocyte–macrophage colony-stimulating factor (GM-CSF) and CCL17 reduced with fenebrutinib treatment (Fig. 5A). A similar profile was observed with the nonselective BTK inhibitor ibrutinib (Fig. 5A). Next, we assessed the ability of fenebrutinib to modulate NLRP3 activity in human iMicroglia using the conventional two-hit method, which involves a priming step with LPS, followed by an activation step with nigericin and measurement of subsequent IL-1β release [58]. Fenebrutinib had no effect on IL-1β release, in contrast to the selective NLRP3 inhibitor MCC-950, which reduced IL-1β levels in a dose-dependent manner (Fig. 5B). Breakdown of the myelin sheath is a common pathological feature in MS [59]. Thus, we measured the impact of fenebrutinib on the ability of human iMicroglia to phagocytose myelin using a pH-sensitive probe. Fenebrutinib had no effect on myelin phagocytosis, while ibrutinib reduced myelin phagocytosis at 1 μM only (Fig. 5C). Therefore, fenebrutinib treatment had minimal effects on TLR4 signaling, NLRP3 signaling and myelin phagocytosis in human iMicroglia.

## Discussion

Neuroinflammation driven by detrimental microglial activity is thought to contribute to disease progression in MS. Chronic active lesions such as paramagnetic rim lesions and slowly expanding lesions are more frequently found in progressive forms of MS, associated with worse disease outcomes and characterized by a rim of morphologically activated microglia and macrophages, which may be loaded with iron [17, 60, 61]. The functional consequences of this innate immune activation are still being elucidated; however, it has been shown that iron accumulation in microglia can induce oxidative stress [62] and contribute to ferroptosis, an iron-dependent form of cell death [63, 64]. Interestingly, a recent study reported increased BTK immunoreactivity at the rims of chronic active lesions compared with inactive lesions, which additionally correlated with iron positivity in microglia and macrophages [20]. While BTK transcript levels were not elevated in MS lesions in the snRNA-seq dataset, increased BTK immunoreactivity was observed in HLA-DR^+^ microglia and macrophages of active MS lesions. Using an MS lesion snRNA-seq dataset to assess levels of innate immune pathway genes previously linked to BTK signaling, we found that certain FcγR-encoding genes, including *FCGR2A* and *FCGR2B*, were significantly upregulated in active and chronic active lesions. This aligns with findings from a recent study that reported increased expression of *FCGR2A* and *FCGR2B* in active and chronic active lesions in individuals with progressive MS [19].

Our findings in diverse human cellular systems, including iMicroglia monocultures, brain tricultures and IMHBOs, indicate that BTK inhibition via fenebrutinib blocks microglial FcγR signaling and its downstream effects. Results from two different immunoassay methods showed that fenebrutinib attenuated the release of multiple proinflammatory cytokines and chemokines from FcγR-activated iMicroglia. This included cytokines such as TNF-α and GM-CSF, which are known to induce neurite loss in vitro [65], and chemokines, including CCL3 and CCL4, which were identified as pharmacodynamic markers of fenebrutinib activity in the periphery [66, 67]. BSG/EMMPRIN, an inducer of MMPs, was another microglial secreted factor modulated by fenebrutinib, and the BSG pathway was the top annotated gene set in the RNA-seq analysis from human brain tricultures. MMPs can impair BBB integrity and neuronal survival [47, 68, 69]. MMP levels are elevated in the CSF and blood of individuals with MS [70, 71] and correlate with magnetic resonance imaging activity in relapsing MS [72]. While endothelial cells were not present in our brain triculture system and could not be assessed, these data raise the possibility that microglial FcγR activation may contribute to BBB breakdown and neuronal damage via release of MMPs; further investigation is warranted. Depending on the cellular system and insult used, BTK inhibition via fenebrutinib also ameliorated neuronal damage in vitro. Prolonged (10 days) exposure of iNGN2 to conditioned media from FcγR-activated iMicroglia increased neurite damage but did not when conditioned media from fenebrutinib-treated iMicroglia was used. FcγR activation in IMHBOs did not induce NfL release, likely due to the acute 24-h exposure to Agg IgG; longer incubation times may be required. In contrast, LPC induced significant NfL release in IMHBOs, which was reduced with fenebrutinib treatment. Since the extracellular TNF-α cytokine concentrations were very low in stimulated IMHBOs, the mechanism by which fenebrutinib blocks LPC-mediated neuronal damage is unlikely to be driven by effects on cytokine release alone. Interestingly, RNA-seq analysis highlighted a striking similarity in gene expression changes in IMHBOs following FcγR activation and LPC exposure. Similar to findings in FcγR-activated brain tricultures, inflammatory pathways were significantly elevated in IMHBOs following FcγR activation and LPC exposure. Downregulation of cholesterol and lipid metabolism (e.g. SREBP) pathways was also observed in response to both stimuli. Cholesterol is produced de novo in the brain and is essential for brain function, as it is one of the main structural components of cell membranes and myelin [73]. SREBPs are a family of transcription factors that regulate expression of genes involved in cholesterol biosynthesis and efflux [74]. Deficient cholesterol metabolism and efflux in microglia and macrophages impair remyelination [75], and TREM2 has been identified previously as a key regulator of microglial cholesterol metabolism [76]. Investigating the molecular mechanism(s) by which BTK inhibition modulates microglial lipid pathways could be one avenue for future research.

The current finding that BTK inhibition via fenebrutinib can block FcγR activation in human microglia is consistent with evidence from peripheral monocytes and macrophages, which shows that myeloid FcγR signaling is BTK dependent. It is unclear if the molecular and cellular phenotypes downstream of BTK-dependent FcγR signaling in microglia resemble those in peripheral innate immune cells. Reports of in vitro microglial FcγR-mediated cytokine and chemokine secretion are similar to observations in peripheral macrophages and align with our findings [24]. In another study, intracerebral injection of immune complexes induced FcγR-dependent inflammation and neuronal damage [77]. However, other investigators found that generation of myelin oligodendrocyte glycoprotein autoantibodies in rodents induces FcγR- and BTK-dependent microglial proliferation without a strong inflammatory or neurotoxic response [78]. Van der Poel et al. [79] also reported that IgG immune complexes require a second proinflammatory signal to induce significant cytokine release in ex vivo primary human microglia. In the same study, the authors detected antimyelin IgGs in postmortem brain tissue of individuals with MS, and others have also identified myelin-specific autoantibodies in the serum or CSF of individuals with MS [80, 81]. Differences in species, in vitro versus in vivo microglial responses and FcγR activation paradigms may account for conflicting data across these studies. Nevertheless, this combined work suggests that the presence of IgG autoantibodies in brains of people with MS may induce damaging microglial responses via BTK-dependent FcγR activation. We considered other innate immune pathways previously linked to BTK function and observed minimal effects of fenebrutinib on TLR4 signaling in human microglia. This aligns with previous work in LPS-stimulated rodent microglia treated with BTK inhibitors [18]. Furthermore, although BTK has been proposed as a regulator of NLRP3 activity [29], fenebrutinib had no effect on microglial IL-1β release induced via the conventional two-hit method of NLRP3 activation via LPS and nigericin stimulation. In the MS lesion snRNA-seq dataset, most representative genes of the TLR and NLRP3 pathways were not overexpressed in the microglia of active lesions. Finally, BTK inhibition has been linked to modulation of macrophage and microglial phagocytosis [4, 18, 82]. While a decrease in myelin phagocytosis was observed with ibrutinib, fenebrutinib did not have an effect, indicating that BTK inhibitor selectivity may influence microglial phagocytosis of myelin. In summary, our data indicate that fenebrutinib blocks the deleterious effects of microglial FcγR activation while preserving other innate immune cell functions.

There are a number of limitations to our current study. Firstly, although bulk RNA-seq analysis of human brain tricultures and IMHBOs captured clear transcriptomic signatures of fenebrutinib activity, this method precludes us from determining cell type-specific gene expression changes with fenebrutinib treatment. Single-cell RNA-seq may help to address this question, even though its use is technically challenging in these miniaturized human cell systems. Secondly, while RNA-seq analyses identified inflammation-related gene sets in both human brain tricultures and IMHBOs, we observed minimal overlap at the single-gene level between these cell systems, which may reflect differences in cell type composition, maturity and 2D versus 3D conditions. Finally, until further data emerge from clinical studies, modeling of microglial responses to BTK inhibitors in acute or subacute in vitro experiments may not fully predict microglial activity in chronic CNS disease settings.

**Fig. 6:**
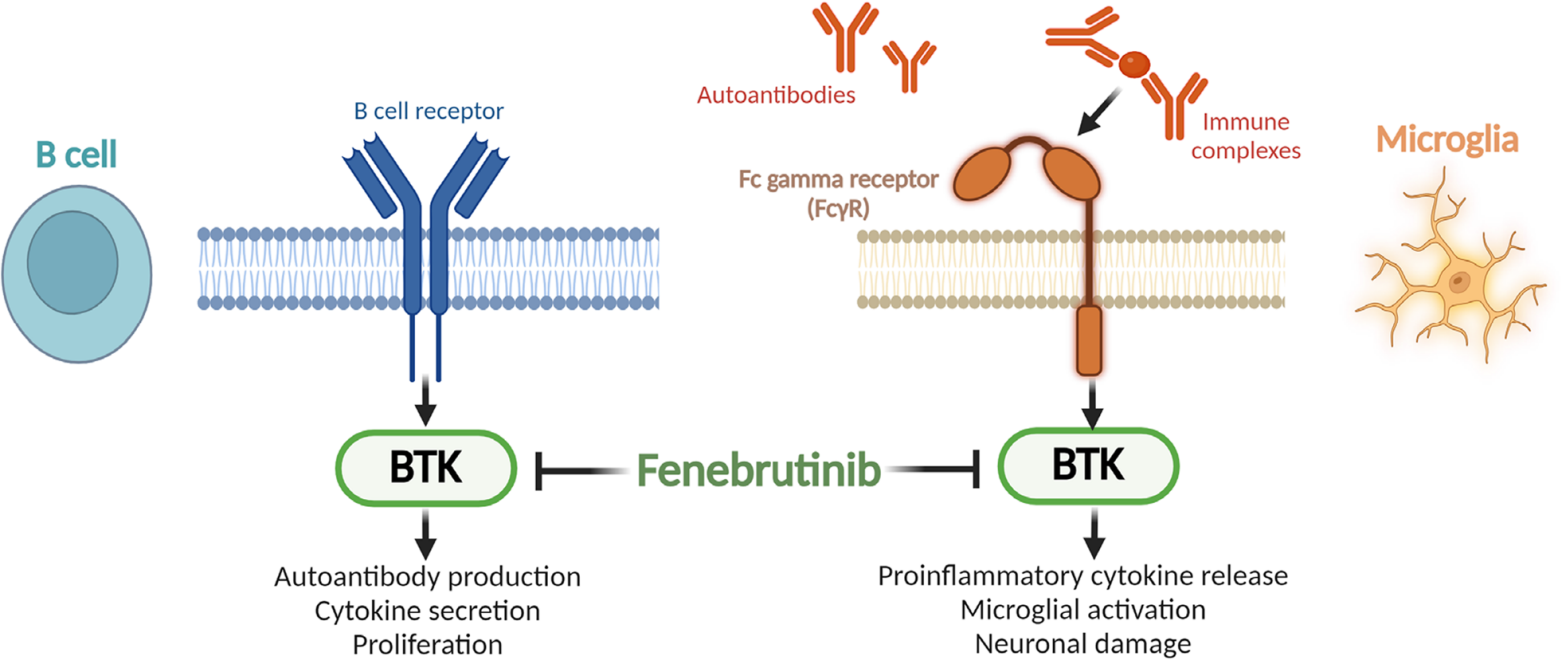
Schematic representation of fenebrutinib effects on both B-cell and microglial pathways. *BTK* Bruton’s tyrosine kinase

In summary, our work increases the understanding of BTK functions in human microglial signaling relevant to MS pathogenesis. Since BTK is not required for the survival of mature B cells [83], BTK inhibitors may preserve humoral immunity in contrast to anti-CD20 B-cell-depleting therapies. Along with increased brain penetrance of small molecules and the potential to directly modulate both B-cell and myeloid cell activity in the CNS and periphery, BTK inhibitors may represent a differentiated therapeutic approach in MS. In support of this, a recent Phase II study in relapsing MS found that fenebrutinib was brain penetrant at clinically relevant concentrations and reduced new brain lesion activity [84]. In addition to its effects on B-cell functions, our human cell data suggest that BTK inhibition via fenebrutinib could act on microglia to attenuate pathogenic neuroinflammation associated with FcγR activation (Fig. 6) and may therefore help reduce progressive neurodegeneration that results from chronic CNS inflammation in MS. Further biomarker and efficacy assessments in clinical trials may help confirm the clinical relevance of this effect on microglia.

## Supplementary Information

Supplementary Material 1.

## Data Availability

Bulk RNAseq raw and count data from brain triculture and brain organoid experiments is available in NCBI GEO under accession number GSE276803.

## Abbreviations

Agg IgG: Heat-aggregated immunoglobulin G
BBB: Blood–brain barrier
BSG/EMMPRIN: Basigin
BTK: Bruton’s tyrosine kinase
CMM: Complete Maintenance Medium
CNS: Central nervous system
CSF: Cerebrospinal fluid
ELISA: Enzyme-linked immunosorbent assay
FcγR: Fc gamma receptor
GFAP: Glial fibrillary acidic protein
GFP: Green fluorescent protein
GM-CSF: Granulocyte–macrophage colony-stimulating factor
HLA-DR: Human leukocyte antigen-DR isotype
iAstrocyte: Induced pluripotent stem cell-derived astrocyte
Iba1: Ionized calcium-binding adaptor molecule 1
Ig: Immunoglobulin
IL: Interleukin
IMHBO: Immunocompetent human brain organoid
iMicroglia: Induced pluripotent stem cell-derived microglia
iNGN2: Neurogenin 2-inducible neuron
iPSC: Induced pluripotent stem cell
LPC: Lysolecithin
LPS: Lipopolysaccharide
MBP: Myelin basic protein
MCM: iMicroglia-conditioned media
MMP: Matrix metalloproteinase
MS: Multiple sclerosis
NF: Neurofilament
NfL: Neurofilament light chain
NLRP3: NACHT, LRR and PYD domains-containing protein 3
NSC: Neural stem cell
PBS: Phosphate-buffered saline
PDL: Poly-d-lysine
PFA: Paraformaldehyde
pwPPMS: People with primary-progressive multiple sclerosis
pwRMS: People with relapsing multiple sclerosis
RNA: Ribonucleic acid
snRNA-seq: Single-nuclei ribonucleic acid sequencing
SREBP: Sterol regulatory element binding protein
TLR: Toll-like receptor
TNF: Tumor necrosis factor
