# Supplementary material for: Fenebrutinib, a Bruton’s tyrosine kinase inhibitor, blocks distinct human microglial signaling pathways
Source: J Neuroinflammation. 2024 Oct 27;21:276. doi: 10.1186/s12974-024-03267-5 (PMC11514909; doi:10.1186/s12974-024-03267-5)
Supplement: Supplementary file 1 — Supplementary Material 1. [file 12974_2024_3267_MOESM1_ESM.docx]

This supplementary file has been provided by the authors to give readers additional information about the study.

Supplement to: **Julie Langlois, Simona Lange, Martin Ebeling, et al.**

**Fenebrutinib, a Bruton’s tyrosine kinase inhibitor, blocks distinct human microglial signaling pathways**

**Contents:**

**Supplementary Fig. 1:** Uncropped Western blot images related to Fig. 2A.

**Supplementary Fig. 2:** Quantification of phosphorylated BTK (pBTK) levels following fenebrutinib treatment in immobilized IgG-stimulated human iMicroglia.

**Supplementary Fig. 3:** TNF-α release from human iMicroglia treated with fenebrutinib (1 μM) and stimulated with immobilized IgG (300 μg/mL) for 24 hours.

**Supplementary Fig. 4:** *Left:* TNF-α release from human iMicroglia stimulated with immobilized IgG, immobilized Fab fragment or soluble Fab fragment (300 μg/mL) for 24 hours. *Right:* Phosphorylated SYK (pSYK) levels normalized to total SYK levels in human iMicroglia stimulated with aggregated IgG (1 mg/ml) for 30 minutes.

**Supplementary Fig. 5:** Supernatant NfL levels (*left*) and neurite density (pan-NF signal; *right*) in Agg IgG-stimulated brain tricultures after 10 days.

**Supplementary Table 1:** Index of reagents

**Supplementary Table 2:** Organoid media components

***
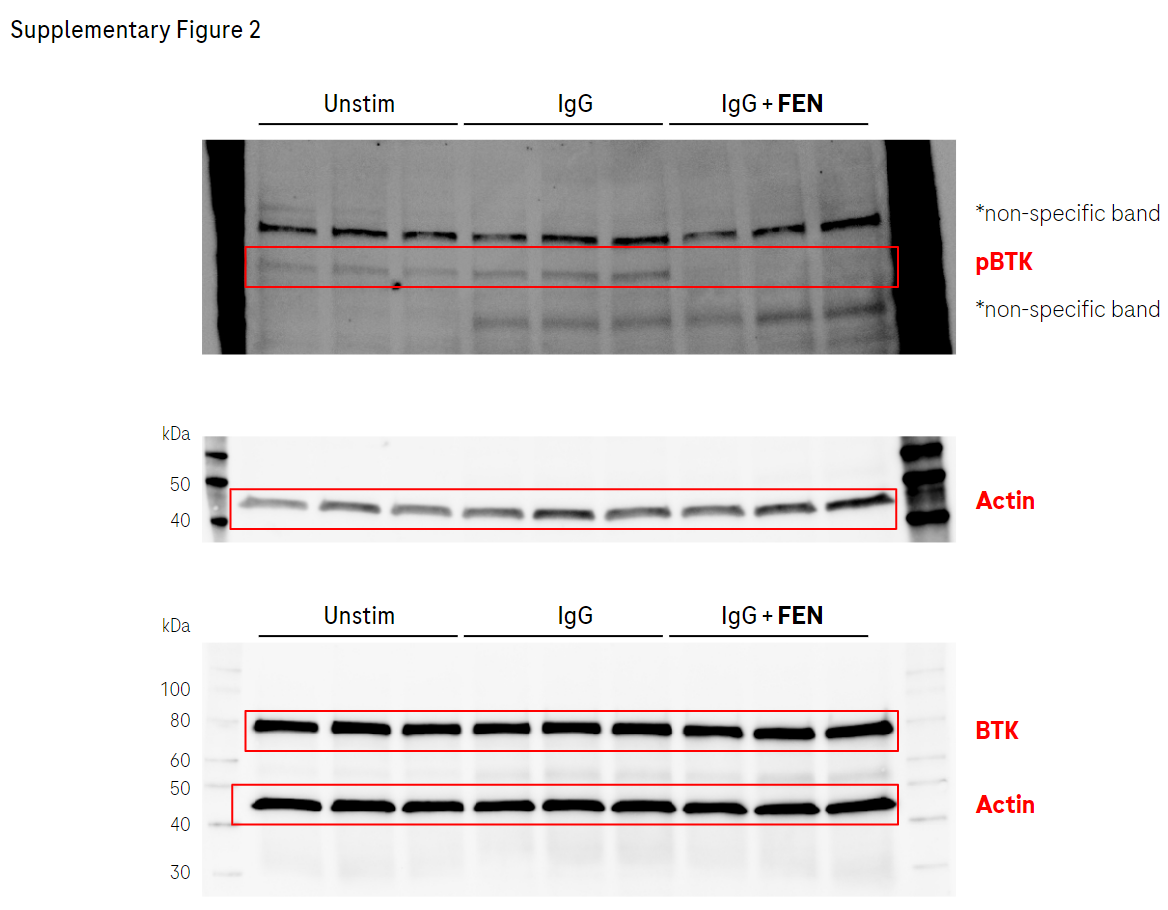
***

**Supplementary Fig. 1:** Uncropped Western blot images related to Fig. 2A.

Western blot of protein lysates from human iMicroglia incubated with fenebrutinib (1 μM) and stimulated with immobilized IgG (300 μg/mL) for 30 minutes. Fenebrutinib treatment reduced immobilized IgG-induced BTK activation as shown by pBTK levels.

BTK, Bruton’s tyrosine kinase; FEN, fenebrutinib; IgG, immunoglobulin G; iMicroglia, induced pluripotent stem cell–derived microglia; pBTK, phosphorylated Bruton’s tyrosine kinase.

***
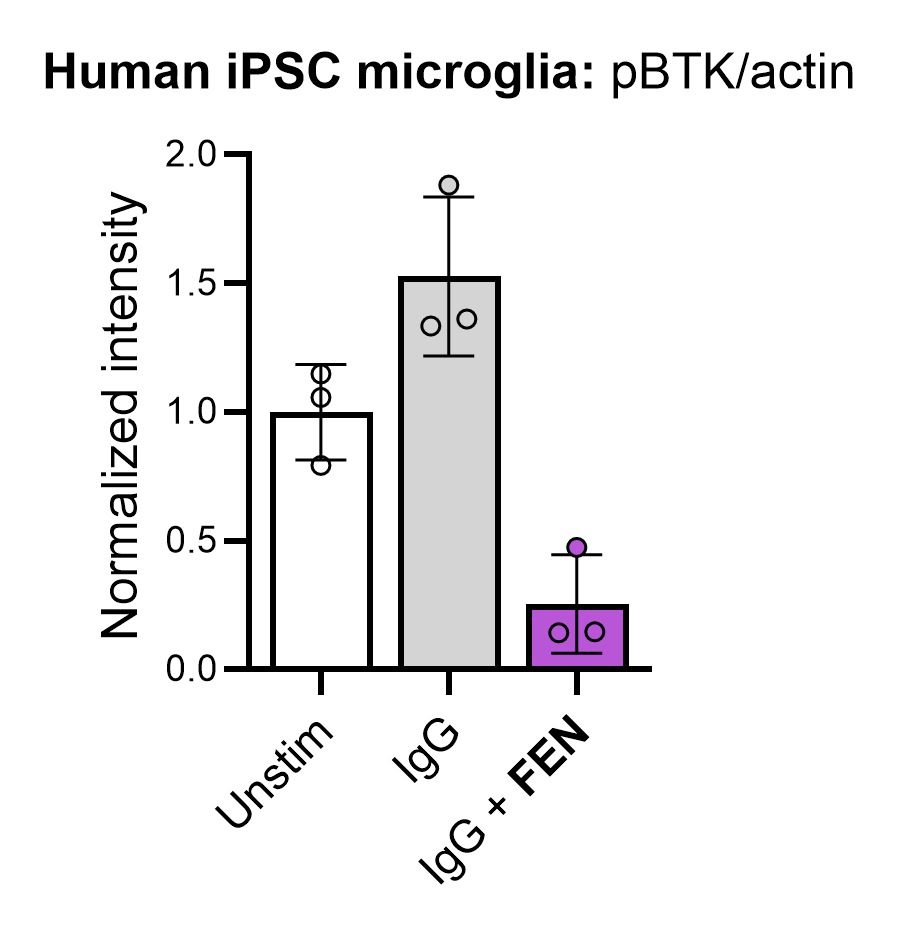
***

**Supplementary Fig. 2:** Quantification of phosphorylated BTK (pBTK) levels following fenebrutinib treatment in immobilized IgG-stimulated human iMicroglia. Data are shown as mean ± SD, with three replicates per condition.

BTK, Bruton’s tyrosine kinase; FEN, fenebrutinib; IgG, immunoglobulin G; iMicroglia, induced pluripotent stem cell–derived microglia; iPSC, induced pluripotent stem cell; pBTK, phosphorylated Bruton’s tyrosine kinase; SD, standard deviation; unstim, unstimulated.

**
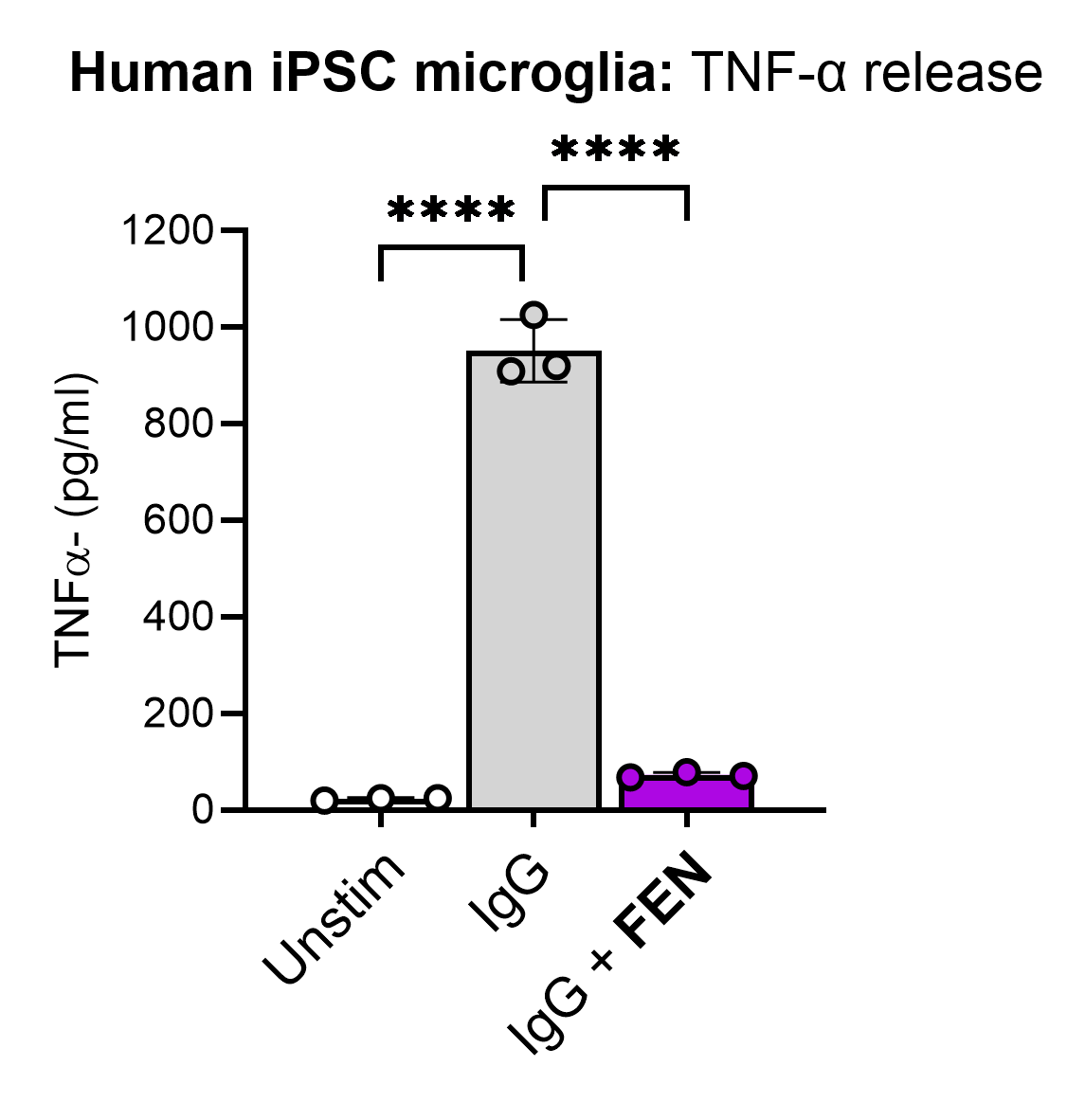
**

**Supplementary Fig. 3:** TNF-α release from human iMicroglia treated with fenebrutinib (1 μM) and stimulated with immobilized IgG (300 μg/mL) for 24 hours. Data are shown as mean ± SD, with three replicates per condition. Significance is indicated by ****P< 0.0001, determined by one‐way ANOVA and Tukey’s post hoc test.

ANOVA, analysis of variance; FEN, fenebrutinib; IgG, immunoglobulin G; iMicroglia, induced pluripotent stem cell–derived microglia; iPSC, induced pluripotent stem cell; SD, standard deviation; TNF, tumor necrosis factor; unstim, unstimulated.


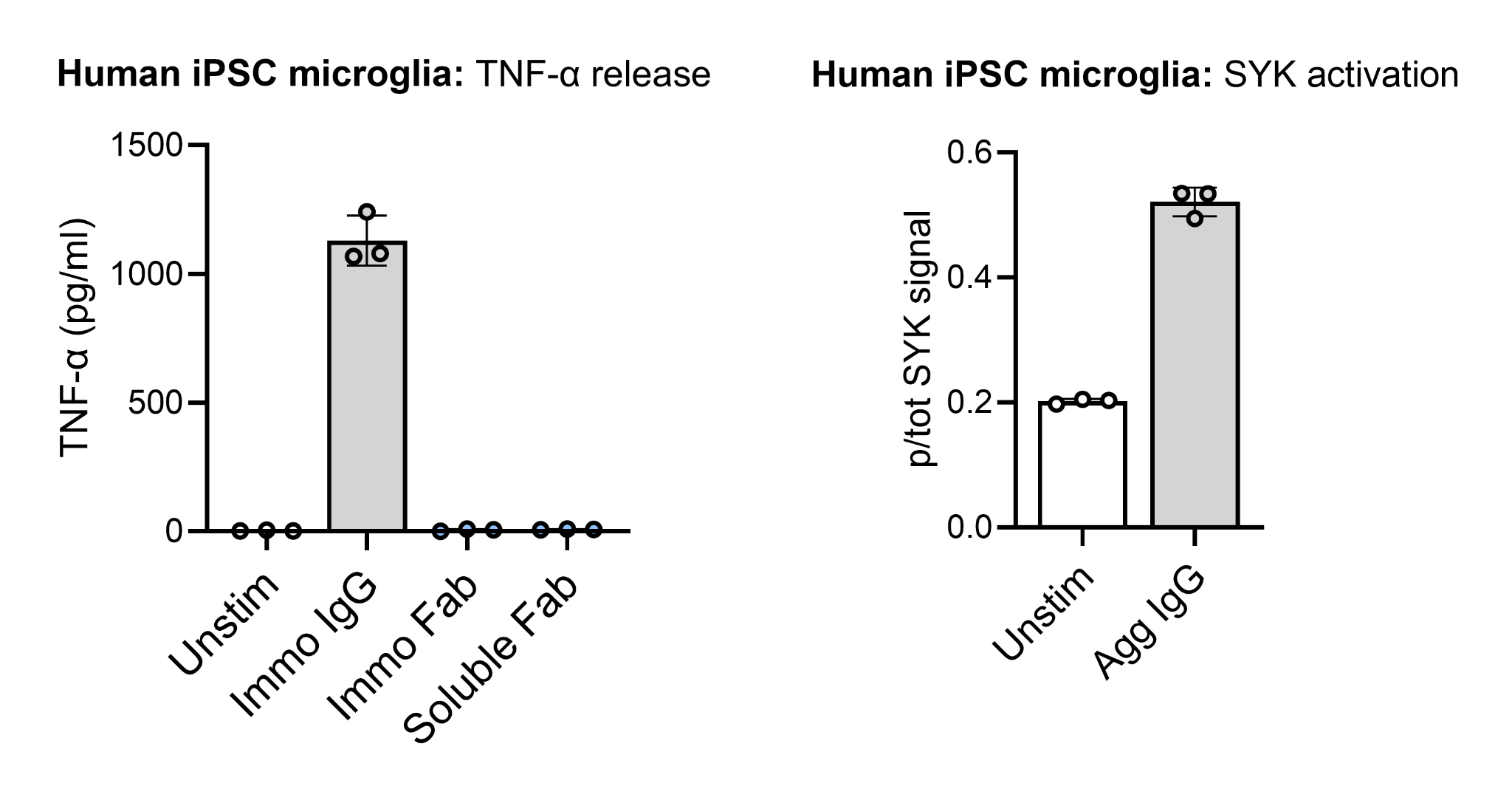


**Supplementary Fig. 4:** *Left:* TNF-α release from human iMicroglia stimulated with immobilized IgG, immobilized Fab fragment or soluble Fab fragment (300 μg/mL) for 24 hours. *Right:* Phosphorylated SYK (pSYK) levels normalized to total SYK levels in human iMicroglia stimulated with aggregated IgG (1 mg/ml) for 30 minutes. Data are shown as mean ± SD, with three replicates per condition.

Agg IgG, heat-aggregated immunoglobulin G; Fab, fragment antigen-binding region; IgG, immunoglobulin G; iMicroglia, induced pluripotent stem cell–derived microglia; immo, immobilized; iPSC, induced pluripotent stem cell; pSYK, phosphorylated spleen tyrosine kinase; SD, standard deviation; SYK, spleen tyrosine kinase; TNF, tumor necrosis factor; unstim, unstimulated.


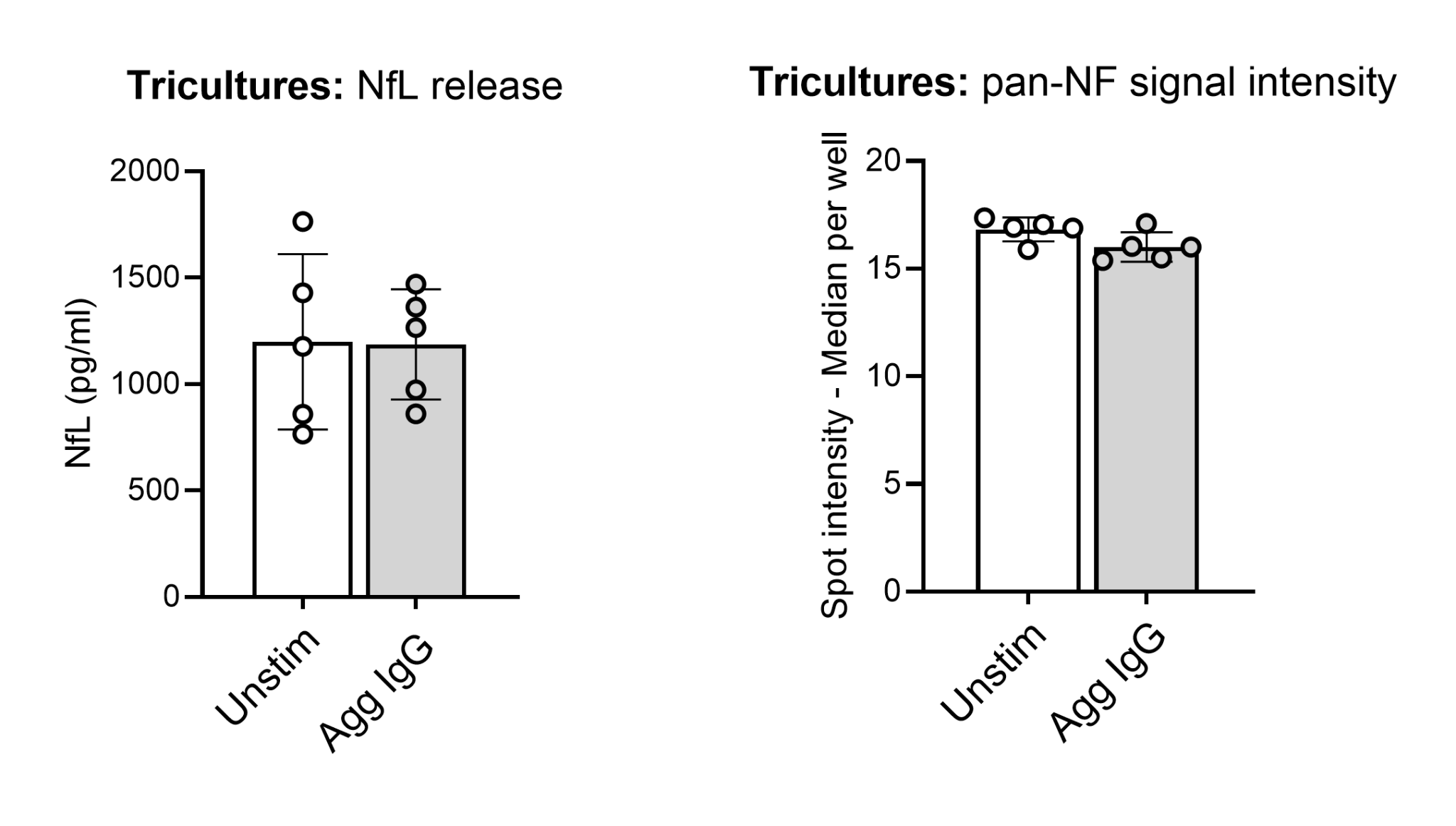


**Supplementary Fig. 5:** Supernatant NfL levels (*left*) and neurite density (pan-NF signal; *right*) in Agg IgG-stimulated brain tricultures after 10 days. Data are shown as mean ± SD, with five replicates per condition.

Agg IgG, heat-aggregated immunoglobulin G; NF, neurofilament; NfL, neurofilament light chain; SD, standard deviation; unstim, unstimulated.

**Supplementary Table 1:** Index of reagents

| **Components** | **Suppliers** | **Catalog numbers** |
| --- | --- | --- |
| Human iPSC-derived microglia | Fujifilm Cellular Dynamics Inc | R1131 |
| Human iPSC-derived astrocytes | Fujifilm Cellular Dynamics Inc | R1092 |
| iCell Microglia Complete Maintenance Medium:   - 50 ml iCell Glial Base Medium - 0.5 ml iCell Microglia Supplement A (100x) - 0.5 ml iCell Microglia Supplement B (100x) - 1 ml iCell Microglia Supplement C (50x) | Fujifilm Cellular Dynamics Inc | - M1054 - M1036 - M1037 - M1055 |
| Neurogenin 2-inducible neurons | StemBANCC |  |
| 96-well plate | Thermo Fisher | 165305 |
| Dendritic polyglycerol amine | Dendrotek | DND400.50-10 |
| Laminin | Sigma | L2020 |
| Neurobasal medium | Gibco | 21103-49 |
| B27 supplement w/o vitamin A (50x) | Thermo Fisher | 12587010 |
| GlutaMax | Thermo Fisher | 35050061 |
| Penicillin-streptomycin | Thermo Fisher | 15140122 |
| Brain-derived neurotrophic factor | Invitrogen | RP-8642 |
| Doxycycline | Sigma | D9891-1G |
| ROCK inhibitor (Y-27632) | Tocris | 1254 |
| Laminin | Roche | 11243217001 |
| Ara-C | Sigma | C6645-25MG |
| Human IgG | Sigma | I4506 |
| Human isotype Fab fragment | In-house | n/a |
| PDL-coated 24-well plates | Greiner | 662940 |
| PDL-coated 96-well plates | Greiner | 655946 |
| LPS from E.coli, Serotype O55:B5 | Enzo Life Sciences | ALX-581-013 |
| Fenebrutinib | In-house | n/a |
| Ibrutinib | MedChemExpress | HY-10997 |
| MCC-950 (CP-456773 sodium salt) | Selleckchem | S7809 |
| DMSO | Sigma | D2650 |
| Nigericin | Invivogen | tlrl-nig-5 |
| Human IL-1β homogeneous time-resolved Fluorescence kit | Cisbio | 62HIL1B2PEG |
| Human XL Cytokine Array kit | R&D Systems | ARY022B |
| LEGENDplex human Inflammation Panel 1 | BioLegend | 740809 |
| LEGENDplex human ProInflammatory Chemokine Panel 1 | BioLegend | 740985 |
| LEGENDplex human ProInflammatory Chemokine Panel 2 | BioLegend | 741158 |
| Human TNF-ɑ homogeneous time-resolved fluorescence kit | Cisbio | 62HTNFAPEG |
| Human TNF-ɑ Quantikine High Sensitivity ELISA | R&D Systems | HSTA00E |
| NfL ELISA kit | Uman Diagnostics | 20-8002 |
| 48-well plate | Costar | 3548 |
| RLT Buffer | Qiagen | 79216 |
| RNeasy Mini kit | Qiagen | 74104 |
| nCounter Human Neuroinflammation Panel | Nanostring | 115000230 |
| Normal goat serum | Thermo Fisher | PCN5000 |
| Anti-Iba1 antibody (1:500) | Fujifilm Wako Pure Chemical Corporation | 019-19741 |
| Anti-GFAP antibody (1:500) | Sigma | AB5541 |
| Anti-pan-neurofilament antibody (1:500) | BioLegend | 837904 |
| Anti-chicken IgG antibody (1:1000), Alexa488 | Thermo Fisher | A11039 |
| Anti-mouse IgG antibody (1:1000), Alexa647 | Thermo Fisher | A21235 |
| Anti-rabbit IgG antibody (1:1000), Alexa568 | Thermo Fisher | A143157 |
| DAPI | Thermo Fisher | 62248 |
| RIPA buffer | Sigma | R0278 |
| cOmplete™ Protease Inhibitor Cocktail | Roche | 11697498001 |
| PhosSTOP | Roche | 4906845001 |
| Pierce BCA Protein Assay kit | Thermo Fisher | 23225 |
| 4-20% precast polyacrylamide gels | BioRad | 4561095 |
| PVDF membranes | BioRad | 1704156 |
| EveryBlot blocking buffer | BioRad | 12010020 |
| Rabbit anti-phospho-BTK (1:100) | Abcam | ab68217 |
| Rabbit anti-BTK (1:1000) | Cell Signaling Technology | D3H5 |
| Mouse anti–β-actin conjugated with HRP (1:5000) | Abcam | ab20272 |
| Alpha SureFire® Ultra™ Multiplex phospho-SYK (Tyr525/526) + Total SYK assay kit | Revity | MPSU-PTSYK-K500 |
| OptiPlate 384-well | Revity | 6007290 |

Agg IgG, heat-aggregated immunoglobulin G; BCA, Bicinchoninic acid; BTK, Bruton’s tyrosine kinase; DAPI, 4′,6-diamidino-2-phenylindole; DMSO, dimethyl sulfoxide; Fab, fragment antigen-binding region; GFAP, glial fibrillary acidic protein; HRP, horseradish peroxidase; Iba1, ionized calcium-binding adaptor molecule 1; IgG, immunoglobulin G; iPSC, induced pluripotent stem cell; IL, interleukin; LPS, lipopolysaccharide; NfL, neurofilament light chain; PDL, poly-D-lysine; phospho-BTK, phosphorylated Bruton’s tyrosine kinase; phospho-SYK, phosphorylated spleen tyrosine kinase; PVDF, polyvinylidene fluoride; RIPA, radioimmunoprecipitation assay; ROCK, Rho-associated protein kinase inhibitor; SD, standard deviation; SYK, spleen tyrosine kinase; TNF, tumor necrosis factor; unstim, unstimulated.

**Supplementary Table 2:** Organoid media components

| **BOM** | | | |
| --- | --- | --- | --- |
| **Components** | **Supplier catalog number** | | **Composition** |
| Neurobasal | ThermoFisher  21103049-049 | | 480 mL |
| B27 | ThermoFisher  17504-044 | | 10 mL |
| P/S+Glutamine | Gibco  10378-016 | | 5 mL |
| GlutaMax | Gibco  35050-061 | | 5 mL |
| BDNF (add fresh) | Peprotech  AF-450-02 | | 50 uL (0.1mg/mL) |
| GDNF (add fresh) | RnD Systems  212-GD-050 | | 50 uL (0.1mg/mL) |
| **coBOM** | | | |
| Neurobasal | ThermoFisher  21103049-049 | | 480 mL |
| B27 | ThermoFisher  17504-044 | | 10 mL |
| P/S+Glutamine | Gibco  10378-016 | | 5 mL |
| GlutaMax | Gibco  35050-061 | | 5 mL |
| BDNF (add fresh) | Peprotech  AF-450-02 | | 50 uL (0.1mg/mL) |
| GDNF (add fresh) | RnD Systems  212-GD-050 | | 50 uL (0.1mg/mL) |
| mCSF (add fresh) | Miltenyi  130-096-493 | | 250 uL (50ug/mL) |
| **NBM** | | | |
| DMEM/F12 | Thermo Fisher Scientific  31331028 | | 500 mL (1:1 w/ neurobasal) |
| Neurobasal | ThermoFisher  21103049-049 | | 500 mL (1:1 w/ DMEM/F12) |
| Gibco B27 supplement w/o Vitamin A (50x) | Thermo Fisher Scientific  12587010 | | 2 mL (1:500) |
| Gibco N2 supplement (100x) | Thermo Fisher Scientific  17502048 | | 10 mL (1:100) |
| Beta-mercaptoethanol (50 uM) | Thermo Fisher Scientific  31350010 | | 1 mL (1:1000) |
| Pen Strep  (10.000 U/mL) | Thermo Fisher Scientific  15140122 | | 10 mL (1:100) |
| FGF (0.1 mg/mL) | Peprotech AF-100-18B | | 1 mL (1:10'000) |
| EGF (0.1 mg/mL) | R&D 236-EG-01M | | 1 mL (1:10'000) |
| BDNF (0.2 mg/mL) | Peprotech AF-450-02 | | 1 mL (1:10'000) |
| **ScaleS4** | | | |
| **Components** | **Supplier**  **catalog number** | **Concentrations** | **Weight/volume per 50 mL** |
| D-Sorbitol | Sigma-Aldrich  S3889-500G | 40% w/v | 20 g |
| Glycerol | Sigma-Aldrich  G2025-100ML | 10% w/v | 5 g |
| Urea | Sigma-Aldrich  51456-500G | 4 M | 12 g |
| Triton X-100 | Sigma-Aldrich  X100-100ML | 0.2% w/v | 0.1 g |
| DMSO | Sigma-Aldrich  D2438-50ML | 15% v/v | 7.5 mL |
| Ultrapure water | dd Milli-Q | - | 15 mL |

BDNF, brain-derived neurotrophic factor; BOM, brain organoid media; coBOM, co brain organoid media; DMEM, Dulbecco's Modified Eagle Medium; DMSO, dimethyl sulfoxide; EGF, epidermal growth factor; FGF, fibroblast growth factor; GDNF, glial cell line-derived neurotrophic factor; mCSF, mouse stem cell factor; NBM, Neurobasal Medium.
